# An indirect high iodine (^131^I) effective dose used for thyroid ablation in patients with thyroid cancer. Is the method of measurement important?

**DOI:** 10.1002/acm2.12901

**Published:** 2020-06-23

**Authors:** Issa A. Al‐Shakhrah

**Affiliations:** ^1^ Physics Department University of Jordan Amman Jordan

**Keywords:** ^131^I radioiodine, radiation effective dose, differentiated thyroid cancer, maximum permissible dose

## Abstract

**Background:**

Radiation effective dose to the red bone‐marrow, a critical organ in the therapy of differentiated thyroid carcinoma (DTC) with radioiodine‐131 (^131^I), cannot be measured directly. As radioiodine concentration is comparable in blood and most organs, and is believed to be similar in red marrow, the effective dose to the blood seems to be a good first‐order approximation of the radiation effective dose to the hematopoietic system and a better means to quantifying exposure from therapy compared to the total amount of activity administered.

**Purpose:**

We applied four formulas (Lassmann et al (standard) [2008], *Eur J Nucl Med Molecul Imaging*, **35**:1405–1412), (Thomas et al. [1993], *Nucl Med Biol*, **20**:157–162), (Sisson et al. [2003], *J Nucl Med*, **44**:898–903; Ha¨nscheid et al. [2009], *Endocr Relat Cancer*, **16**:1283–1289) and (Ha¨nscheid et al. [2006], *J Nucl Med*, **47**:648–654) and compared between the estimated values of the effective dose that were obtained by three formulas and those obtained by the standard one.

**Materials and methods:**

Twenty‐seven patients, 22 women and 5 men, suffering from DTC were enrolled in this study. Whole‐body probe measurements and blood collections (2 mL whole‐blood samples) were conducted at 2, 6, 24, 48, 72**–**96 h after the administration of ^131^I to obtain time**–**activity curves. Whole‐body measurements were performed as conjugate view (anterior and posterior) counts by scintillation camera imaging.

**Results:**

By comparing the values of blood effective dose that were obtained by applying Thomas et al. [1993], *Nucl Med Biol*, **20**:157–162; Sisson et al. [2003], *J Nucl Med*, **44**:898–903 and Ha¨nscheid et al. [2009], *Endocr Relat Cancer*, **16**:1283–1289, and Ha¨nscheid et al. [2006], *J Nucl Med*, **47**:648–654, techniques, with those obtained by (Lassmann et al (standard technique) [2008], *Eur J Nucl Med Molecul Imaging*, **35**:1405–1412), we found that these values are, respectively, 15.0%, 40.0%, and 41.0% more than those obtained by using the standard method. To our knowledge no papers have been published previously that compare between these dosimetric approaches.

**Conclusion:**

Highly overestimated or highly underestimated results obtained by a certain method or technique, compared with those obtained by the standard method, are not desirable, they tend to exaggerate in applying radiation protection procedures, by increasing or decreasing, which, in both cases, become far from the realistic or recommended procedures. As an operating philosophy, the objective of radiation safety practices simply should not be to keep radiation doses within legal limits or maximum permissible doses (MPD_s_), but to keep them “as low as reasonably achievable” (ALARA concept). MPD_s_ should not be considered as thresholds below which exposure to radiation is of no concern, they are not assumed to be totally risk free, and any reasonable technique for reducing radiation dose may have potential benefits in the long run.

## 1 INTRODUCTION

Blood dosimetry was introduced by Benua et al.^1^ in a study published in 1962. They found that radioiodine therapy is safe if the blood dose is restricted to <2 Gy (200 rad), while keeping the whole‐body retention <4.4 GBq at 48 h, and the pulmonary uptake at 24 h < 3 GBq.^1^, ^2^

Radiation exposure from fixed activities is very heterogeneous. Depending principally on the patient's size and renal clearance, the calculated blood absorbed dose per administered unit of activity can differ by a factor of more than 5.^3^ A low absorbed dose to the blood might predict reduced radioiodine availability for target tissue uptake and, therefore, a low absorbed dose to the target tissue.

Usually 1.1**–**3.7 GBq is prescribed for the first radioiodine therapy after thyroidectomy in newly diagnosed DTC patients to ablate the remaining glandular tissue. Higher amounts of ^131^I are given in subsequent therapies or in cases of metastatic disease. For safety reasons the activity is usually limited to approximately 7.4 GBq.^3^

However, a higher administered activity is usually desired to achieve higher tumor doses. To avoid serious complications, the commonly used dose concept published by Benua et al.^1^ for radioiodine treatment of DTC restricts the blood dose to <2 Gy. In their protocol, measurements of iodine retention in the blood and whole body with a tracer activity are required to estimate the blood dose before the radioiodine therapy. This method has been applied successfully.^4^, ^5^

Several total body dosimetry formulas in the treatment of DTC have been developed and refined in a series of international multicenter trials,^3^, ^6^, ^7^ some of these methods use blood samples, whereas others prefer measuring radiation externally by Geiger Müller or gamma camera. In addition, measurements can be performed at different time intervals.

The aim of this study is to calculate the radiation effective doses in the blood of patients with DTC treated with radioactive iodine using a modified Benua method. To achieve this we employ standard operational procedures (SOP). In addition, we compare between the estimated values obtained by three formulas and those obtained by standard SOP method. To the best of our knowledge, no studies have been published that compare between these dosimetric approaches.

## 2 SUBJECTS, MATERIAL AND METHODS

Twenty‐seven patients, 22 women, and 5 men, suffering from DTC were recruited for this study. All patients provided informed consent to participate in the study.

The information and data concerning these patients (weight, height, retention function, and residence time), are taken from table 3 in the appendix of Ref. [3].

The data extraction was performed by drawing regions of interest (ROI) at each site according to a dosimetry operational manual with detailed instructions that were distributed to all participating centers before the beginning of the study.

Whole‐body probe measurements and blood collections (2 mL whole‐blood samples) were conducted 2, 6, 24, 48, 72**–**96 h after the administration of ^131^I to obtain time**–**activity curves. The “Standard Operational Procedures for Pre‐therapeutic Dosimetry” (SOP) equation based on the generally accepted formalism of the Medical Internal Radiation Dose Committee (MIRD) was used to determine the mean blood absorbed dose, Lassmann et al. (standard).^6^ They applied the following equation:

(1) $$\frac{D_{\mathrm{blood}}}{A_{0}}\left(\frac{\mathrm{Gy}}{\mathrm{GBq}}\right)=108{x\tau }_{\mathrm{ml}\;\mathrm{of}\;\mathrm{blood}}\left(h\right)+\left(\frac{0.0188}{{\left(\mathrm{wt}\left(\mathrm{kg}\right)\right)}^{2/3}}\right)\times \tau_{\mathrm{total}\;\mathrm{body}}\left(h\right)$$

where $\tau_{\mathrm{total}\mathrm{body}}\mathrm{is}$ the total body residence time; $\tau_{\mathrm{ml}\mathrm{of}\mathrm{blood}}$ is the residence time in a milliliter of whole blood. Finally wt is the patient’s weight in kg.

A method to estimate blood dose from external whole‐body counting without blood sampling was proposed by Thomas et al.^8^

The following equation was applied:

(2) $$\frac{D_{\mathrm{blood}}}{A_{0}}\left(\frac{\mathrm{mGy}}{\mathrm{MBq}}\right)=\left(\frac{15.12}{\mathrm{BLV}\left(\mathrm{ml}\right)}+\frac{0.0188}{{\left(\mathrm{wt}\left(\mathrm{kg}\right)\right)}^{2/3}}\right)\times \tau_{\mathrm{total}\;\mathrm{body}}\left(h\right)$$

Sisson et al.^9^ and Ha¨nscheid et al.^10^ proposed to use the 48 h whole‐body retention measured in a diagnostic assessment to adapt the activity in the subsequent radioiodine therapy in case of markedly low or high 48 h whole‐body uptake, and they applied the following formula:

(3) $$\frac{D_{\mathrm{blood}}}{A_{0}}\left(\frac{\mathrm{mGy}}{\mathrm{MBq}}\right)=-\left(\frac{15.12}{\mathrm{BLV}\left(\mathrm{ml}\right)}+\frac{0.0188}{{\left(\mathrm{wt}\left(\mathrm{kg}\right)\right)}^{2/3}}\right)\times \frac{t\left(h\right)}{\ln\left(R\left(t\right)\right)}$$

The individual blood volume (BLV) can be estimated from the patient’s weight wt (kg) and height ht (cm), by applying the following formula that was proposed by Retzlaff et al.^11^:

Where BLV = 31.9 × ht + 26.3 × wt − 2402 for males and BLV = 56.9 × ht + 14.1 × wt − 6460 for females.

Furthermore, a blood dose estimate from a single measurement of the whole‐body retention can be deduced if the retention R(*t*) at *t* hours after the radioiodine administration is taken to be representative for the total‐body residence time.

The absorbed dose to the blood was calculated with a modified method deduced from a procedure originally described by Thomas et al.^8^ This refined method was applied by Ha¨nscheid et al.^3^ They applied the following equation:

(4) $$\frac{D_{\mathrm{blood}}}{A_{0}}\left(\frac{\mathrm{mGy}}{\mathrm{MBq}}\right)=116{x\tau }_{\mathrm{ml}\;\mathrm{of}\;\mathrm{blood}}\left(h\right)+\left(\frac{0.104}{{\left(\mathrm{wt}\left(\mathrm{kg}\right)\right)}^{0.86}}\right)\times \tau_{\mathrm{total}\;\mathrm{body}}\left(h\right)$$

This technique is based on the formalism by the MIRD Committee of the Society of Nuclear Medicine. Published S values,^12^, ^13^, ^14^ were used to account for contributions of activity in the blood and the remainder of the body to the blood dose.

Blood effective dose estimates calculated according to the techniques of Thomas et al.^8^ (Sisson et al.,^9^ Ha¨nscheid et al.^10^) and Ha¨nscheid et al.,^3^ were compared with those obtained by Lassmann et al. (standard technique).^6^

## 3 RESULTS

**Fig. 1:**
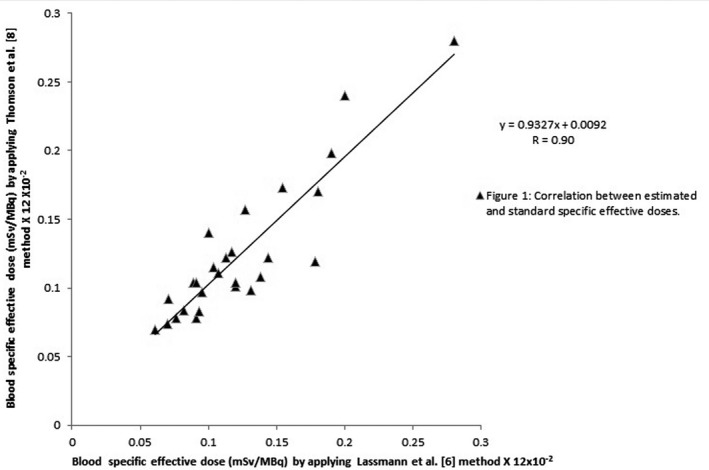
Correlation between the values of radiation‐specific effective doses obtained by applying the standard method (Lassmann et al. (standard)),^6^ and those by applying Thomas et al.^8^ method

We used the regression equation to analyze our results. We found that the correlation coefficient (r) between the results that were obtained by applying the Lassmann et al. (standard method),^6^ and those that were obtained by applying Thomas et al. method,^8^ is equal to 0.9 as shown in Fig. 1, which is considered as an excellent correlation.

**Fig. 2:**
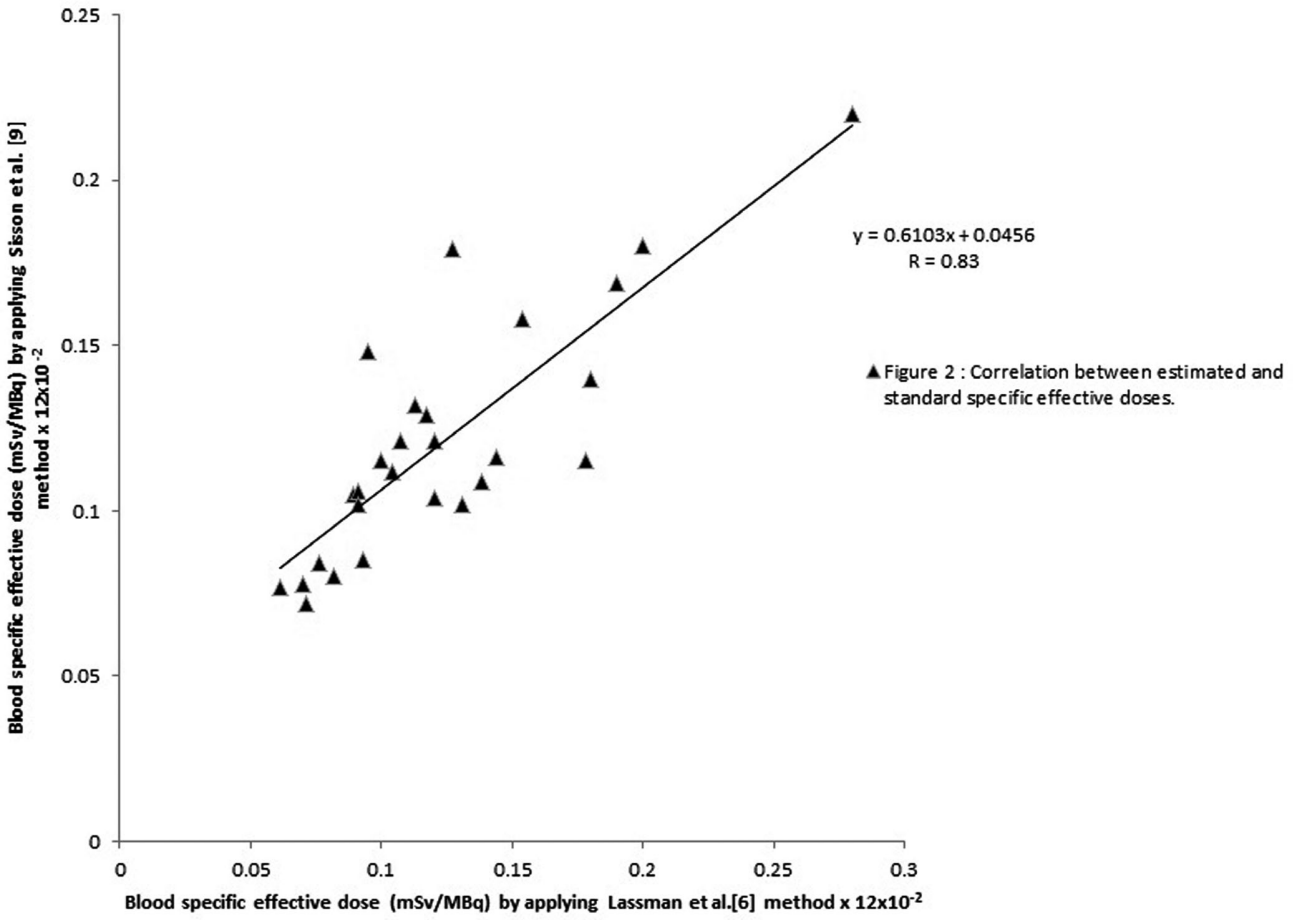
Correlation between the values of radiation‐specific effective doses obtained by applying the standard method (Lassmann et al. (standard)),^6^ and those by applying Sisson et al.^9^ method

**Fig. 3:**
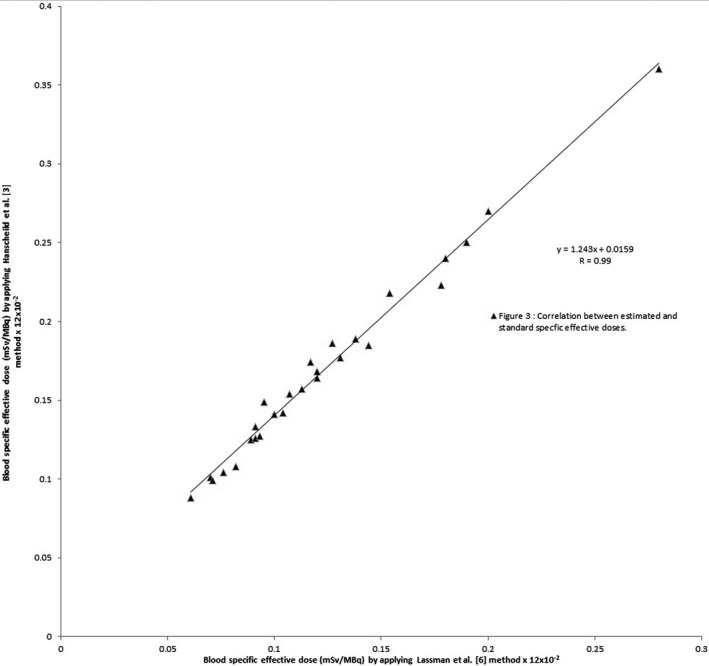
Correlation between the values of radiation‐specific effective doses obtained by applying the standard method (Lassmann et al. (standard)),^6^ and those by applying Ha¨nscheid et al.^3^ method

The results obtained by applying the Sisson et al.^9^ and Hänscheid et al.^10^ method demonstrated that 11 out of 27 cases (40.7%) have underestimated values of effective dose, whereas the remaining 16 cases (59.3%) have overestimated values of effective dose, with a very good correlation coefficient (r = 0.83) as shown in Fig. 2, compared with those obtained by the Lassmann et al. (standard technique).^6^ The results also show that the values that were calculated by applying the Ha¨nscheid et al.^3^ technique are all highly overestimated, which is not realistic, even though they have an excellent correlation (r = 0.99), as shown in Fig. 3 with the standard value. Highly overestimated or highly underestimated results obtained by a certain method or technique are not desirable, they tend to exaggerate in applying radiation protection procedures, by increasing or decreasing, which, in both cases, become far from the realistic or recommended procedures. We believe that the results obtained using the method of Thomas et al.^8^ are better than the corresponding ones obtained using the methods of Sisson et al.^9^, Hänscheid et al.^10^ and Hänscheid et al.^3^. They are more realistic (66.7% of the cases are overestimated) and have excellent correlation (r = 0.9) compared with those obtained by standard value.

Blood (bone‐marrow), specific absorbed dose (mG/MBq), specific effective dose (mSv/MBq), and effective dose (mSv) estimated values that were obtained by applying the four methods for a female patient (70 kg mass), who was administered an amount of 4440 MBq for thyroid ablation are shown in Table 1.

**Table 1:** Blood (bone‐marrow)‐specific absorbed dose (mGy/MBq), specific effective dose (mSv/MBq), and effective dose (mSv) estimated values that were obtained by applying four methods, for a female patient (70 kg mass), who was administered with 4440 MBq for thyroid ablation.

Method	Specific absorbed dose (mGy/MBq)	Specific effective dose (mSv/MBq)	Administered activity (MBq(mCi))	Effective dose (mSv)
Lassmann et al.^6^	0.100	0.0120	4440 (120)	53.28
Thomas et al.^8^	0.115	0.0136	4440 (120)	61.27
Sisson et al.^9^	0.140	0.0168	4440 (120)	74.59
Ha¨nscheid et al.^3^	0.141	0.0169	4440 (120)	75.12

Our estimated values for specific effective dose (mSv/MBq) and effective dose (mSv) for adult subjects from selected internally administered radiopharmaceuticals are shown in Table 2 *.*

**Table 2:** Specific effective dose (mSv/MBq) and effective dose (mSv) estimates to adult subjects from selected internally administered radiopharmaceuticals.

Radiopharmaceutical	Specific effective dose (mSv/MBq)^29^	Administered activity (MBq)	Effective dose (mSv)
^99m^Tc‐MDP	6.1 × 10^−3^	740 (20)	4.51
^99m^Tc‐DTPA	8.2 × 10^−3^	185 (5)	1.52
^18^F‐FDG	3.0 × 10^−2^	296 (8)	8.88
^67^Ga‐ citrate	1.1 × 10^−1^	185 (5)	20.35

DTPA, diethylenetriaminepentaacetic acid; FDG, fluorodeoxyglucose; MDP, methylene diphosphonate.

## 4 DISCUSSION

From a historical point of view, it has long been accepted that a single administration of a higher radioiodine level results in a more successful ablation. This was based on the hypothesis that larger levels of radioiodine, are more likely to ablate remnants and destroy residual micrometastases than lower levels.^15^

Leila et al.^16^ investigated whether higher activities of administered ^131^I would necessarily increase the absorbed dose to the blood in treating patients with DTC. The study revealed that absorbed dose to the blood of patients with DTC administered with 5.55 GBq ^131^I is significantly higher than that of patients administered with 3.7 GBq of ^131^I. However, there is no significant difference in the absorbed dose to patients’ blood when treated with 7.4 GBq of ^131^I compared to 5.55 GBq. Given that the absorbed dose to the blood is a better predictor of ablation success than overall ^131^I administered,^17^ these findings suggest that 5.55 GBq would be the most favorably administered activity compared to 3.7 GBq. In addition, 7.4 GBq of ^131^I in thyroid ablation administered activity of 5.55 GBq is not only more advantageous therapeutically, but also causes fewer therapeutic problems than a dose of 7.4 GBq.

A patient may be released from the medical facility either when the activity levels in the patient drop below 1,110 MBq or when dose rates at 1 m from the patient drop below 50 mSv per hour. The Office of Nuclear Regulatory Research (NRR) recommends 1,221 MBq or a dose rate at 1 m of less than 70 mSv per hour for ^131^I. When either criterion is met the patient may be released to return home ^18^.

Effective half‐life for ^131^I is 5 days. In the first 24 h after dosing, patients received the therapy excrete of 30%**–**75% of the administered dose.^19^, ^20^ Most of it is in the urine, but a significant amount enters the gastrointestinal tract via salivary excretion and gastric secretion.

The effective half‐life depends on the physical and biological half‐lives and varies among patients.^21^, ^22^ The absorbed dose varies and is proportional to the effective half‐life. In patients for whom the effective half‐life is shorter than assumed in a protocol, and therapy could result in undertreatment or the need for a second treatment. If a patient has an effective half‐life longer than assumed, the patient will receive a higher absorbed dose than planned and be exposed to unnecessary radiation.

With high‐dose therapy, the dose to the blood should be <200 rad to reduce bone‐marrow toxicity. The total whole‐body retained dose at 48 h should be less than 4.44 GBq in widespread metastatic thyroid carcinoma and 2.96 GBq in the presence of lung metastases^23^, ^24^; the latter is a precaution to avoid pulmonary fibrosis.

For patients, being treated as outpatients has social benefits. If recommended guidelines for releasing patients are followed, and if patients' living conditions are assessed adequately, outpatient treatment with high‐doses of ^131^I is safe, cost effective, and improves patient satisfaction.^25^

The release of patients treated with radioactive ^131^I from hospital remains a controversial issue as a result of the range of guidelines implemented by national regulatory bodies responsible for radiation protection in various countries worldwide. In 2016, the South African Department of Health, Directorate: Radiation Control added conditions (numbers 50 and 90) for licences to be used as radioactive nuclides. These conditions state that the patients must be hospitalized when the dose rate at 1 m is above 25 μSv/h, or more than 555 MBq of iodine‐131 was administered to the patient. The results from the literature have shown that in setting patient release criteria (PRC), several countries have considered the socioeconomic conditions prevailing in their countries to achieve harmony between public protection and cost associated with hospitalization.^26^

The effective dose was introduced by the International Commission on Radiological Protection (ICRP),^27^, ^28^ as an attempt to characterize a nonuniform internal dose by a single number. This quantity was intended primarily for estimating radiation risks and doses received by radiation workers, although its extension to clinical nuclear medicine studies has been supported by the ICRP. The effective dose represents the whole‐body dose that would result in the same overall risk as the nonuniform dose distribution actually delivered. This is achieved by assigning different weighting factors to the doses delivered to individual organs.

$$\begin{array}{cc} \text{Effective dose}\left(\text{mSv}\right) & =\text{Absorbed dose}\left(\text{mGy}\right)\\ & \;\times \text{Radiation weighting factor}\left(W_{R}\right)\\ & \;\times \text{Tissue weighting factor}\left(W_{T}\right) \end{array}$$

where W_R_ = 1 for all radiations (γ and β) used in diagnostic nuclear medicine (imaging procedures) and therapeutics (for thyroid ablation).

W_T_ = 0.12 for blood (bone‐marrow).

Table 1 shows that there are differences between the values that were obtained from the four methods.

Comparing the values of blood effective dose that were obtained by applying Thomas et al.,^8^ Sisson et al.,^9^ Ha¨nscheid et al.,^10^ and Ha¨nscheid et al.^3^ methods, with those obtained by Lassmann et al. (standard technique),^6^ we found that these values are, respectively, 15.0%, 40.0%, and 41.0% more than those obtained by using the standard one.

All the values of blood effective dose that were obtained by the four methods are far less than the maximum permissible blood effective dose.

Maximum permissible dose (mSv) = 2000 mGy (2 Gy) × 1 × 0.12 = 240 mSv.

National Council on Radiation Protection and Measurement (NCRP) and Nuclear Regulatory Commission (NRC) regulations, have recommended as an operating strategy or philosophy that the objectives of radiation safety practices should not simply be to keep radiation doses below maximum permissible doses (MPD), but to keep them “as low as reasonably achievable” (ALARA concept).

In our laboratory, we prefer applying the Thomas et al.^8^ method, for determining the blood effective dose, because it can estimate blood dose from external whole‐body counting without blood sampling. The activity which is administered to the patients for thyroid ablation is ranged between 1.11 and 7.4 GBq with an average of 4.44 GBq.

Errors or uncertainties from measurements can be reduced by careful and repeated measurements, using reliable instruments and properly calibrating the instruments.

## 5 CONCLUSION

From the three methods applied in this research, we believe that the estimated values (results) that are obtained by Thomson et al.^8^ are better than those obtained by the other two methods such as Sisson et al.,^9^ Hänscheid et al.^10^ and Hänscheid et al.^3^ They are more realistic (66.7% of the cases are overestimated) and have excellent correlation coefficient (r = 90%) compared with those obtained by Lassmann et al. (the standard method)^6^). Highly overestimated or highly underestimated results obtained by certain methods or techniques, compared with those obtained by the standard method, are not desirable, as they tend to exaggerate in applying radiation protection procedures, by increasing or decreasing, which, in both cases, become far from the realistic or recommended procedures. As an operating strategy or philosophy, the objective of radiation safety practices should not be simply to keep radiation doses within legal limits, but to keep them “as low as reasonably achievable” (ALARA concept).

## CONFLICT OF INTEREST

The author declares that there is no conflict of interest that could be perceived as prejudicing the impartiality of the research reported.

## FUNDING INFORMATION

This research did not receive any specific grant from any funding agency in the public, commercial, or not‐for‐profit sector.
